# The Co‐Structuring of Gesture‐Vocal Dynamics: An Exploration in Karnatak Music Performance

**DOI:** 10.1111/cogs.70137

**Published:** 2025-11-30

**Authors:** Lara Pearson, Thomas Nuttall, Wim Pouw

**Affiliations:** ^1^ Department of Music, Max Planck Institute for Empirical Aesthetics; ^2^ Institute of Musicology, Faculty of Arts and Humanities, University of Cologne; ^3^ Music Technology Group, Pompeu Fabra University; ^4^ Department of Computational Cognitive Science, Research Center for Cognitive Science and Artificial Intelligence Tilburg University; ^5^ Donders Institute for Brain, Cognition, and Behaviour, Radboud University

**Keywords:** Vocal performance, Gesture, Multimodality, South Indian music, Motion tracking, Dynamic time warping, Data dashboard

## Abstract

In music performance contexts, vocalists tend to gesture with hand and upper body movements as they sing. But how does this gesturing relate to the sung phrases, and how do singers’ gesturing styles differ from each other? In this study, we present a quantitative analysis and visualization pipeline that characterizes the multidimensional co‐structuring of body movements and vocalizations in vocal performers. We apply this to a dataset of performances within the Karnatak music tradition of South India, including audio and motion tracking data of 44 performances by three expert Karnatak vocalists, openly published with this report. Our results show that time‐varying features of head and hand gestures tend to be more similar when the concurrent vocal time‐varying features are also more similar. While for each performer we find clear co‐structuring of sound and movement, they each show their own characteristic salient dimensions (e.g., hand position, head acceleration) through which movement co‐structures with singing. Our time‐series analyses thereby provide a computational approach to characterizing individual vocalists’ unique multimodal vocal‐gesture co‐structuring profiles. We also show that co‐structuring clearly reduces degrees of freedom of the multimodal performance such that motifs that sound alike tend to co‐structure with gestures that move alike. The current method can be applied to any multimodally ensembled signals in both human and nonhuman communication, to determine co‐structuring profiles and explore any reduction in degrees of freedom. In the context of Karnatak singing performance, the current analysis is an important starting point for further experimental study of gesture‐vocal synergies.

## 1 Introduction

Across a range of performance contexts worldwide, vocalists tend to gesture with hand and head movements while they sing. Such gesturing can be understood as part of the performer's expressive interaction with the audience and also with the music itself (Davidson, 2001). Qualitative studies have noted correspondences between gestures and musical motifs (Pearson, 2013; Rahaim, 2012), but systematic analyses of this relationship remain few. In this study, we focus on relationships between sung vocalization and co‐occurring gesture, seeking to characterize the multidimensional co‐structuring of vocalizations and body movement and investigate how this varies across performers. We thereby focus on *multimodal co‐structuring*, which is constrained by a range of physiological and aesthetic “control parameters” that structurally reduce the degrees of freedom of the multimodal performance.

We explore this in the context of a South Indian tradition known as Karnatak music (Karṇāṭaka Saṅgīta), which has a strong emphasis on vocal performance (Saṅgīta can be translated as “sung together”) and where vocalists typically gesture with hand and head movements as they sing (see https://youtu.be/INk1KvYOf8U).^1^ The tradition originated in the royal courts and temples of South India and is still performed today in concert halls and at temple festivals. In both this and related North Indian vocal styles, the majority of performers gesture spontaneously while singing, producing simultaneous strands of body movement and sound. Their gestures are neither planned ahead nor based on any formal system (Rahaim, 2012), but rather they are spontaneous and unreflective in a way that is similar to much of our co‐speech gesturing (Cooperrider, 2019; Gallagher, 2005). However, as with co‐speech gesturing (Cooperrider, 2019; Feyereisen, 2017), certain gestural forms and tendencies are picked up through enculturation, and so some similarities can be seen across performers (Rahaim, 2012).

Discussion of co‐singing gesture can be found in the growing body of work on gesture in Indian music contexts, largely focusing on the North Indian, Hindustani style (Clayton et al., 2023; Fatone et al., 2011; Leante, 2009; Mani, 2017; Paschalidou, 2017; Paschalidou et al., 2016; Pearson & Pouw, 2022; Rahaim, 2012), in addition to research on gesture in Western art music and choral contexts (Brunkan & Bowers, 2021; D'Amario et al., 2023; Nafisi, 2013; Prové, 2022), as well as in popular music (Davidson, 2001; 2006). Definitions of gesture in this body of work vary, so we should, therefore, clarify the concept of gesture used in this article. We draw here on Kendon's definition of gesture as “visible action when it is used as an utterance or as part of an utterance,” where utterance is defined as “any ensemble of action that counts for others as an attempt by the actor to ‘give’ information of some sort” (2004, p. 7). As a vocal performance is clearly “utterance” in that it aims to give information (e.g., on expressive interpretation, phrasing, or other melodic and lyrical features), Kendon's definition of gesture works well for our context of Karnatak singing.

While providing a detailed definition of gesture is not the goal of this study, we can highlight that a definition needs to accommodate a diverse array of meaning‐making processes in which hand movement and posturing play a role. This includes indexing of very basic biomechanical interactions (Pouw & Fuchs, 2022), as well as deixis, iconicity, and all the other modes already described by gesture studies (metaphoric, emblematic, etc.). As we find similar meaning‐making strategies present in both co‐speech and co‐singing gestures (such as biomechanics, deixis, and iconicity), this definition needs to be inclusive of the hand movements and postures produced during vocal performance. Notably, the functions of speech and song substantially overlap (Cross, 2015; Cummins, 2020), and so this similarity in multimodal meaning‐making strategies is unsurprising.

Although it may be useful in some cases to categorize gestures by function or semiotic mode, in our previous work (Pearson & Pouw, 2022), we deliberately avoided this and instead made a plea for thinking about gestures as naturally entangling a variety of often simultaneous meaning‐making processes. We continue with the same approach in this study. A forceful gesture serves not only to produce a biomechanical impulse on the respiratory‐vocal system, or only to show an iconic quality of force that aligns with the vocal performance, but rather, they can do both. Furthermore, they can entangle other meaning‐making processes; for example, a similar forceful hand movement directed toward another performer may also have a deictic component in addition to its metaphoric or biomechanic consequences. Our standpoint here is not idiosyncratic. Recent modern annotation schemes like M3D already account for the simultaneity of different functions of gestures, where, for example, iconic gestures may have a beat‐like dimension too (Rohrer et al., 2025). We would go one step further and suggest that the idea of strict orthogonal (i.e., independent) dimensions fails to fully capture the ecological scenario, and that instead there is an aesthetic entanglement between different gestural functions that determines the character of the multimodal performance in a more holistic and nondecomposable way (see Pearson & Pouw, 2022). These definitions, principles, and observations form the basis of our concept of gesture.

This article presents a quantitative analysis and visualization pipeline for characterizing the multidimensional co‐structuring of body movement and vocalizations in vocal performers. This characterization is gradient, in the sense of providing a *continuous* measure of (dis)similarity across several dimensions of interest in gestures (e.g., position, acceleration) and vocalizations (e.g., change in f0, change in amplitude). We apply this pipeline to a dataset of 3.79 h of Karnatak (South Indian) vocal performances (audio, video, motion‐capture), from three expert performers, investigating how performer gestures (hand and head movements) co‐structure with short musical patterns, referred to here as motifs. The Karnatak music term most closely related to motif is *sañcāra*, with both terms referring to short musical phrases that have a sense of coherence. Such units are musically meaningful in Karnatak music, acting as building blocks of compositions and extemporizations (Viswanathan, 1977). Indeed, motifs are of structural significance in most musical styles, and are a common focus of research in both music analysis and cognition (Eitan & Granot, 2009; Zbikowski, 1999).

In this observational study, we ask whether there is a systematic relationship between the sonic similarity of motifs and the kinematic similarity of the co‐occurring gestures. In addition, we seek to better characterize the multidimensional codependencies of body movement and vocalization, asking how the various sonic and kinematic features examined (sonic features: f0, Δf0, loudness, spectral centroid; gesture kinematics: 3d position, velocity, acceleration of both hand and head motion) differ in the extent to which they co‐structure. A further key question concerns the differences between individual performers’ co‐structuring of sound and movement.

### 1.1 Background

This research builds on work in gesture studies showing that semantically related gestures move alike (Pouw et al., 2021), where it was found in a two‐part study that silent or co‐speech gestures have similar kinematic trajectories when they convey a similar concept. Specifically, in the study's first part, it was shown that in silent gestures, the word2vec‐based *semantic* distance between the conveyed concepts had a weak but reliable correlation with the dynamic‐time‐warping‐based *kinematic* distance of the silent gestures. In the second part, using a different dataset, it was shown that when two people were communicating complex visual shapes, the semantic distance between the names for different shapes they arrived at by the end of the conversation were correlated with the kinematic distance between gestures they produced for the respective shapes during the conversation. This study provides an important grounding for the idea that gestures, while often highly unconventionalized, are structurally interrelated such that their kinematic differences can be informative about semantics (and vice versa). In fact, Pouw et al. (2021) argued from this that gestural semantics may have similar contextual constraints that ground meaning as the principle of distributional semantics in text, where we can glean semantic dissimilarity between words simply by assessing differences in the context with which such words structurally associate. Hagoort and Özyürek (2024) have recently further explored this idea of distributed gesture semantics suggested in Pouw et al. (2021), demonstrating its current interest among researchers.

In the current study, we draw on the design and findings of Pouw et al. (2021) to hypothesize that in singing, vocal motifs constrain the co‐occurring gesture kinematics, and that motifs that sound more alike will tend to co‐occur with gestures that move more alike. We test this on a dataset of Karnatak vocal performances. While preliminary investigations were already reported in Pearson, Nuttall, and Pouw (2023), recent research using 2D video‐based tracking has provided additional evidence that bodily gestures and vocal performances indeed co‐pattern to the extent that three stereotypical melodic motifs chosen by the researchers could be distinguished based on the degree of kinematic distance between the concomitant gesture (Nadkarni et al., 2023). However, the question of whether gestures structurally interrelate with vocal performance in general (rather than in only three motifs) requires examination with a wider range of more freely varying vocal units and high‐resolution kinematic analysis, the approach taken in the present article. In addition, the analyses presented below afford insight into which precise dimensions of gestures co‐structure with which vocal modality, and how this varies across individual performers. Through this broader approach, involving a very wide range of different motifs, found using machine learning methods rather than chosen by the authors, we also aim to problematize the conceptual juxtaposition of vocalizations and gestures as isolated units that are structurally combined from a static library of stereotypical types. As discussed below (see Sections 1.2 and 4.2), we suggest that stereotypical gesture‐vocal combinations, such as circular gestures for vocal oscillations, are sparse events that are indicative of a much wider overall gesture‐vocalization co‐structuring. Instead, therefore, we explore co‐structuring as a gradient phenomenon over a wide range of different motifs, not chosen by the authors, to understand the broader structural coherence of gesture and vocalization in performance.

It is important to note that examining the co‐structuring of gesture and vocalization is not the same as asking whether gesture and vocalization time series or peaks are coupled over time (as in Pearson & Pouw, 2022). For example, it is possible that some particular gestures tend to co‐occur with specific vocal motifs, but that those gestures and their concomitant vocalization are not synchronized in their concurrent activity. Pearson and Pouw (2022) find evidence of gesture‐vocal synchrony, especially for acceleration and f0, whereby acceleration peaks tend to co‐occur with peaks in vocal f0, which are also correlated in their magnitude; a finding recently replicated (Nadkarni et al., 2024). Acceleration is an important kinematic marker of force‐transfers onto the body, and Pearson and Pouw (2022), therefore, suggest that this coupling might be in part biomechanical, in line with research on co‐speech gestures (for an overview, see Pouw & Fuchs, 2022). This previous study also reveals a high degree of performer variability and concludes that while concurrent gesture‐vocal coupling is most stable across performers in the acceleration‐f0 dimension, there are likely to be many more constraints that structure the multimodal performance. One way to explore such constraints is through the analysis of systematic co‐structuring of particular movement qualities with particular vocal qualities, as done in this article.

### 1.2 Recurring and stereotypical gestures

In qualitative research on Indian vocal music practices, connections have been noted between performers’ sung musical phrases and their co‐occurring hand gestures (e.g., Pearson, 2013; Rahaim, 2012). For example, Matthew Rahaim (2012) notes that in one Hindustani vocal performance, 30 out of the 34 occurrences of a particular melodic shift were accompanied by a gesture where the vocalist's hands curl around a small empty space. Rahaim suggests that such recurring gestural patterns can be considered catchments, conceptualized in co‐speech contexts as regions of recurring gestures that index underlying discourse themes (McNeill, 2000). A related concept in co‐speech gesture research is that of recurrent gestures (Harrison & Ladewig, 2022; Ladewig, 2014; Mortimer & Pereira, 2023; Müller, 2018), which show a stable form‐meaning pairing within individual and/or culturally shared repertoires (Müller, 2018, p. 277), implying a stability beyond a single interaction or performance.

While it is clear from existing qualitative studies on Hindustani and Karnatak vocal performance that there are cases where particular vocal motifs repeatedly co‐occur with gestures that have highly similar forms, we suggest that such recurring motif‐gesture combinations may be infrequent relative to the entire set of gesture‐vocal utterances within any given performance. Furthermore, the recurrences may be significantly more gradient and imperfect, such that we should understand them as having a chaotic quality, much like a complex multivariable system visiting similar regions in a space of possibilities but never repeating the exact states (Favela, 2020); or as “repetition without repetition” (Bernstein, 1967). To understand this gradient recurrence at a larger scale, in this article, instead of focusing only on a few stereotypical gesture‐vocal motifs, we explore gesture‐vocalization relations across a wide range of vocal segments, located automatically using machine learning methods rather than chosen by the researchers.

### 1.3 Current study

The research questions and goals of this study, as applied to our Karnatak vocal dataset, are as follows:

- Is there a systematic relationship between the sonic similarity of motifs and the kinematic similarity of the co‐occurring gestures? Based on Pouw et al. (2021), we hypothesize that motifs that sound more alike tend to co‐occur with gestures that move more alike.
- What are the multidimensional codependencies of body movement and vocalizations? That is to say, how do the various sonic and kinematic features examined differ in the extent to which they co‐structure?
- Are both head and hand movements part of the systematic relationship with sonic features, or is there systematicity with vocalization in one movement modality only? We hypothesize that sonic features can be predicted to some extent from kinematic features, with head and hand features combined being more predictive than simply one or the other.
- How does the co‐structuring of sonic and kinematic features vary across different performers? What can we learn about the different ways that individuals co‐structure sound and movement in their performances?

**Fig. 1:**
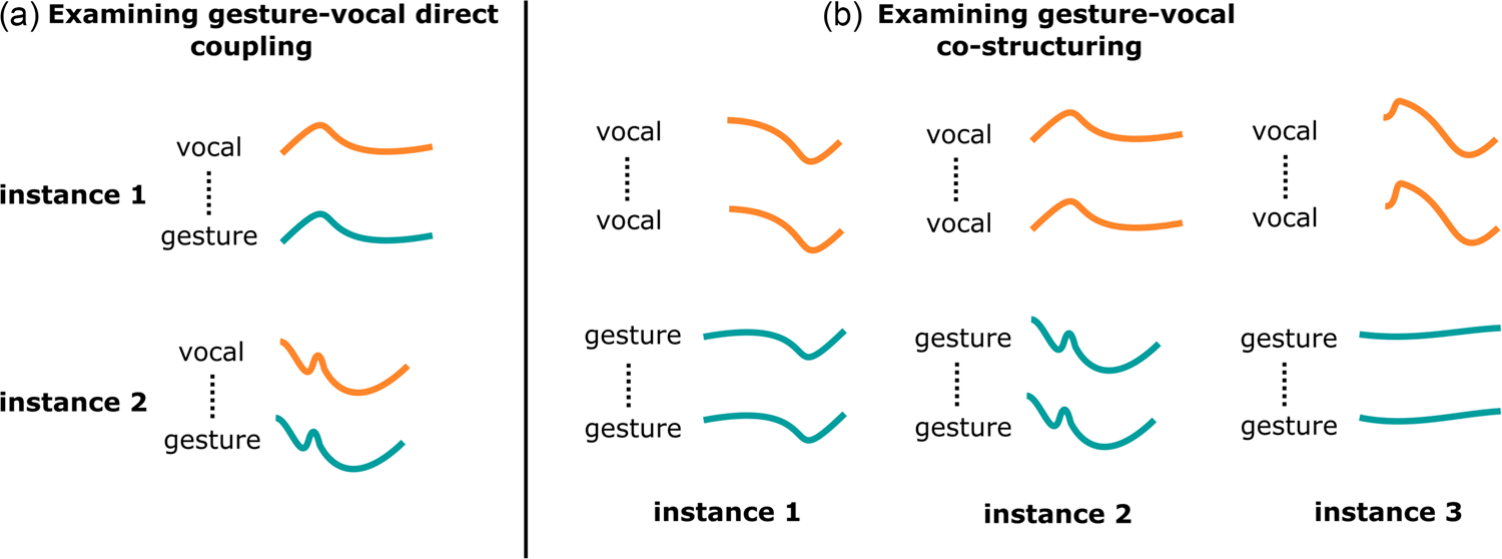
A figure highlighting the schematic difference of direct gesture‐vocal coupling versus gesture‐vocal co‐structuring. Previous research has looked at direct coupling between some time‐varying vocal feature and some time‐varying gesture feature (Nadkarni et al., 2024; Pearson & Pouw, 2022), such that over many instances, there is a statistically reliable association between time series at similar points in time (the left panel shows this direct coupling). In our current study, we instead look at gesture‐vocal co‐structuring (illustrated in the right panel). Gesture‐vocal co‐structuring is a second‐order association (an association between associations), based on vocal motifs that are similar co‐occurring with gestures that are also similar (Pouw et al., 2021). This may mean that they co‐structure because there is some direct coupling (as in the right panel, instance 1), but it may also mean, as in instance 2 and instance 3, right panel, that there is little (instance 2) to no (instance 3) similarity between gesture and a vocalization trajectory. In other words, there is a second‐order relation across the instances where gesture and vocalizations are co‐structuring, which is not to be confused with a first‐order relation, where gesture and vocalizations couple directly. We refer to this second‐order relation as gesture‐vocal co‐structuring.

The research design employed to answer these questions is an observational study based on a dataset of 44 performances by three expert Karnatak vocalists, based in South India, which overlaps with the dataset used in Pearson and Pouw (2022). The synchronized audio and motion capture data, together with animations created from the motion capture data, are made open for the first time with the current publication.^2^ Our analyses aim to quantify the degree to which gestures that are more alike co‐occur with vocalizations that are more alike. It should be emphasized that we do not engage in any direct comparison between gesture kinematics and vocal acoustics (as in Nadkarni et al., 2024; Pearson & Pouw, 2022; Wagner et al., 2014), rather we compare similarity between all possible pairs of gestural features and all possible pairs of vocal features to understand whether gestures that are more similar co‐occur with vocalizations that are more similar. We refer to this as an examination of gesture‐vocal co‐structuring, in the sense that we explore whether they systematically structure in relation to each other. Fig. 1 provides a schematic figure that aims to make intuitive the distinction between gesture‐vocal co‐structuring (our approach in this article), versus direct gesture‐vocal coupling (as in previous studies).

Our approach is gradient in the sense that we compute a measure of co‐structuring that is continuous, factoring in the full range of degrees of similarity between gesture pairs and motif pairs. Our approach enables us to take into account a very broad range of co‐structuring types and causes. For example, while co‐structuring found between pitch and hand position may be due to hand position moving up vertically while vocal pitch moves up (metaphorically), it may also be due to less obvious systematic co‐structuring whereby hand position moves out to the side when pitch moves up (which can often be seen in these performances). It is the systematicity of the co‐structuring that is found through our analysis, without a priori deciding on the nature of that co‐structuring. We view this as a strength of our approach as it opens the analysis to relationships beyond those we might expect due to existing findings on crossmodal correspondences between sound and movement (Eitan & Granot, 2006). Our methods also provide insight into the qualities of any co‐structuring by separating out different kinematic and sonic features, to learn which co‐structure more strongly with each other. Our analyses, therefore, differentiate particular dimensions of co‐structuring, which can be used to provide insight into potential underlying mechanisms as well as to explore differences between performers.

The analysis pipeline reported here contributes to reproducible methods that can be applied to investigate systematic but gradient co‐structuring in many different types of multimodal communication, including music making and other forms of vocal performance, but also in animal multimodal communication (Partan & Marler, 1999). It thereby broadens the scope relative to earlier work on conventionalized speech semantics and gradient gesture kinematics (Pouw et al., 2021).

## 2 Materials and methods

### 2.1 Performers and performances

The study is based on a total of 44 performances by three expert Karnatak vocalists, recorded in South India. All performances are of a Karnatak musical format known as *rāga ālāpana*, which is improvisational in the sense that performers spontaneously play with and manipulate motivic elements that are characteristic of the raga (melodic framework) being performed (Viswanathan, 1977). It should be noted that this format is always sung using nonlexical vocables. The vocalists were requested to sing *ālāpana* for eight different ragas, and they chose their own order of presentation. The total singing time across all performances was 3.79 hours. The singing time in the individual *rāga ālāpana* performances lasted *M* = 310.00 s (*SD* = 118.72), with durations ranging from 100 to 586 s. The three vocalists are all right‐handed (1 male, 2 female; *M*_age_ = 35.7 years, *SD*_age_ = 5.8), and participated in this study having given their informed consent.

The vocalists, based in Chennai and Bengaluru, are highly proficient and currently active performers within the South Indian, Karnatak music community, each having combined studying and performing experience of between 22 and 37 years. They come from different musical lineages, have no history of performing together, and were recorded in different locations on different days. The vocalists were chosen based on their high level of expertise, representativeness of the tradition, and willingness to be recorded for this research. Such decisions were made by the first author, who has expertise in the Karnatak music tradition, with over 16 years of performance and research experience in collaboration with Karnatak musicians.

In the recordings, each vocalist was accompanied by an oscillating drone, called a tambura, which is either a plucked instrument or an electronic simulation of this. All three vocalists recorded solo sessions without violin accompanists, while two of the vocalists additionally performed sessions with a violin accompanist. Where present, the violinist shadows the vocalist, performing mainly in the gaps between the vocalist's phrases, but sometimes overlapping at the ends of phrases. As a result, the vast majority of motifs in our dataset include voice and tambura only. It should be noted that *rāga ālāpana* has neither a musical meter nor a steady beat throughout, and, therefore, entrainment to a repeated steady beat does not arise in this musical format. While there may be some gestural interaction between vocalist and violinist in those sessions where other performers were present, the fact that their phrases overlap rather than being performed together, and our dataset's mixture of solo and accompanied performances means that such interactions are unlikely to have skewed our results in a particular direction.

### 2.2 Measurements and equipment

An inertial measurement system (Xsens MVN Awinda [60 Hz sampling]) was used to track each vocalist's upper body in terms of position, velocity, and acceleration in 3D space (Paulich et al., 2018; Xsens, 2018). From among the recorded body points, the left hand, right hand, and head segments were used for the gesture kinematic analyses. Audio was recorded at 48 kHz using Neumann KM184 condenser microphones placed directly in front of the performers, as in a typical Karnatak concert. Video was recorded with GoPro Hero4 cameras at 50 fps, and these recordings were used for qualitative cross‐checking of our quantitative research. Clapperboards were performed by the vocalist and recorded at the start and end of each performance. Synchronization of the different streams was achieved using the pulse timecode system (Timecode Systems:Pulse, n.d.) and checked manually for peak deceleration at clapperboard closure. The resulting synchronized audio and motion capture data, together with animations created from the motion capture data, are included in the Karnatak raga alapana dataset, made open with this publication: https://osf.io/6huvd/.

### 2.3 Methods

**Fig. 2:**
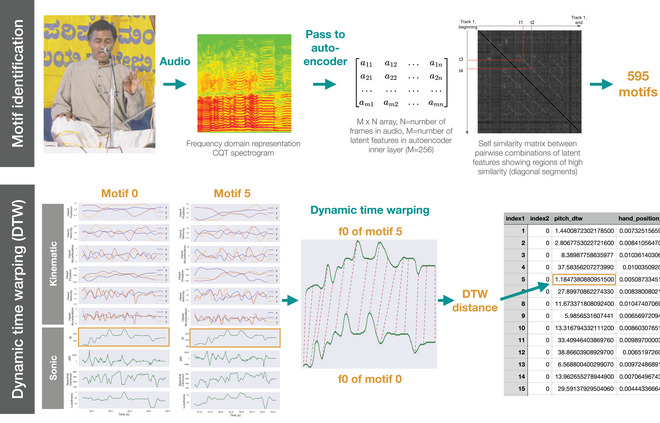
An overview of the first two stages in the analysis pipeline. The upper row shows the motif identification process, wherein pairwise regions of consistently high melodic similarity are identified as repeated motifs using features learnt by an autoencoder. The lower row visualizes the dynamic time warping process, in which DTW distances are calculated for all possible pairs of 10 sonic and kinematic features and placed in the DTW distance dataframe. The photograph shows the Karnatak vocalist, Hemmige S Prashanth, performing on stage in Mangalore in 2014.

Our pipeline begins with the identification of regions in our 44 performances that are likely to represent repeated melodic motifs; these form the basis upon which all subsequent analyses are built. The second part of our pipeline uses dynamic time warping (DTW) to compare the similarity between sonic/kinematic temporal features for each pairwise combination of all motifs (see Fig. 2). DTW quantifies the similarity between two time series by computing a minimum‐cost alignment that accommodates nonlinear temporal distortions (Sakoe & Chiba, 1987). Unlike simple Euclidean distance, DTW allows for flexible warping of the time axis, enabling a meaningful comparison between sequences that may differ due to slight variations in temporal execution, as seen in speech, gesture, and physiological signals. Our study is concerned with understanding whether the similarity (as computed using DTW) between kinematic features correlates with the similarity between sonic features: whether motifs that sound more alike, move more alike. All code necessary to reproduce the analysis and visualizations are provided here: https://github.com/thomasgnuttall/KarnatakGestureVocalCostructuring/tree/main.

#### 2.3.1 Automated repeated melodic motif extraction

We use a machine learning methodology tailored for Karnatak music to locate regions of repeated melodic patterns across the dataset (Nuttall, Plaja‐Roglans, Pearson, & Serra, 2022), implemented as part of the compIAM package and used with its default parameters (Plaja‐Roglans, Nuttall, & Serra, 2023). The process identifies regions of consistent melodic similarity by computing self‐similarity of autoencoder embeddings from constant‐Q transforms of the raw audio. To be returned by the process, patterns have to repeat at least once in a performance and have a minimum length of 1.5 s. From the 44 performances, 595 unique, nonoverlapping regions are identified from 31 of those performances, each region corresponding to a melodic motif that is repeated at least once (see Supplementary Materials, Section S1).

#### 2.3.2 Feature extraction and processing

For each of the 595 motifs, we extract a time series corresponding to the following features: **f0** of the predominant sung melody, using a machine learning methodology tailored for Karnatak music (Plaja‐Roglans, Nuttall, Pearson, et al., 2023), also implemented as part of the compIAM package; **Δf0**, an approximation of the first derivative of the f0 curve as outlined in Keogh and Pazzani (2001); **loudness**, $L=10\;log_{10}\left(\frac{S}{ref}\right)$, where S is the power spectrum of the raw audio signal and ref is its maximum value; the **spectral centroid** of the raw audio signal, a representation of timbre that has been found to correspond to ratings of perceptual salience (Schultz et al., 2021); and the three‐dimensional **position**, **velocity**, and **acceleration** of the hand and head event trajectories as captured using the Xsens MVN Awinda system. Hence, in total, each of the 595 motifs is described by four *sonic* time series (f0, Δf0, loudness, and spectral centroid), and six *kinematic* time series (hand position, head position, hand velocity, head velocity, hand acceleration, and head acceleration)—10 in total. The kinematic time series are three‐dimensional.

The sonic time series features are smoothed using a one‐dimensional Gaussian filter with a standard deviation of 2.5, decided by manual inspection of the time series alongside their audio, where our aim was to retain salient melodic detail, including the subtle ornamentations typical of the Karnatak style. Gaps in the pitch time series of 350 ms or less are linearly interpolated to join short breaks in the melody (absence of pitch information). In Karnatak vocal music, such short breaks often occur within ornamentations, for example, due to glottal closure and other rapid vocal movements that include unpitched sound. While these are arguably real breaks in pitch, they tend not to be perceived as such in real time, and also do not constitute a conceptual break in the motif as understood and sung by the performer. Since we subsequently use DTW on these pitch curves, which does not allow for breaks within the analyzed time series, this interpolation ensures the inclusion of more motifs by filling these short gaps with a reasonable estimate between their source and destination pitch values.

The kinematic time series features are smoothed using a second‐order Savitzky−Golay filter with a window length of 125 ms, again decided by manual inspection of the curves alongside their video to ensure that perceivable movements in the video are still present in the time series data. Each kinematic time series is rotated such that the line between the positional centroid of the left and right shoulders is parallel to the x‐axis—so that the performer is facing the “front of the stage”—and the origin of the gesture space set to the centroid of the performer's pelvis position. For the kinematic hand data, the predominant hand of the performer is selected for each motif based on that which has the most kinetic energy, KE, as computed across the entire duration of the motif: from the velocity curve, v, $KE=\frac{1}{2}\;mv^{2}$, where m is the mass of the body part in question and is assumed equal for both sides. The x‐axis values are mirrored for the right hand such that all motifs occur in the same “left‐hand space.” This allows us to compare gestures that are recurrent but mirrored due to hand preference differences. 89.0% of motifs are identified as left‐handed, and 11.0% are identified as right‐handed. The proportion of motifs with a ratio between the dominant‐hand energy and the nondominant hand energy of greater than 1.2 is 97.0%, indicating that there is almost always a clear dominant hand.

For each pairwise combination of all motifs, the DTW distance is computed between each of their respective features; hence, each motif pair has one DTW value for f0, one for Δf0, one for loudness, and so on. To account for slight variances in segmentation point between pairs of otherwise highly similar motifs, we use a custom, dependent DTW implementation that allows for each extreme (start and end) of the warping path to begin within 0.1L of the start and end of each pattern, where L is the length of the longest motif in the pair. The Sakoe−Chiba window size is also equal to 0.1L; this setting constrains the warping path such that distant points in the query and referent time series are not compared. The resulting dataframe has 176,715 rows (one for each pair of motifs excluding self‐comparisons), and 10 columns containing DTW distances for each of the 10 features.

We validate the use of DTW as a proxy for perceptual similarity of melody by comparing the computed distances with manual annotations of similarity from two Karnatak vocalists (one was a professional performer who sung for our dataset, while the other was a performer with over 15 years of experience, who was completely external to the project) finding a significant correlation between DTW distance and the vocalists’ perception of whether two motifs are the same or different (see also Akamine et al., 2025 for recent corroborating results). The full details of this analysis are included in the Supplementary Materials, Section S7.

#### 2.3.3 Correlation and regression analyses

Two analyses are performed on the dataframe containing the DTW distances of all possible motif pairs for the four sonic features (f0, Δf0, loudness, and spectral centroid) and six kinematic features (hand position, head position, hand velocity, head velocity, hand acceleration, and head acceleration). The accompanying GitHub repository includes the pitch time series of the 595 motifs, the dataframe with metadata and the code to create it, and the analysis results and the code to reproduce them.^3^

**Overview Analysis 1: Do sonic motif DTW distances covary with spatiotemporal patterns of gesture?**

We hypothesize that motifs that sound more alike tend to co‐occur with gestures that move more alike. This analysis is, therefore, aimed at learning whether there is a systematic relationship between sonic similarity of motifs and kinematic similarity of the co‐occurring gesture, that is, whether the similarity between sonic features (sonic DTW distances) correlates with the similarity between kinematic features (kinematic DTW distances). For each pairwise combination of four sonic feature columns and six kinematic feature columns, we compute the Spearman's rank correlation coefficient between the column values. We first do this across all performers, which constitutes 24 (4×6) computations, and then further compute these values on subsets of the DTW distance dataframe corresponding to individual performers. For the “all performer” test, the number of patterns from each is randomly subsampled such that each performer is represented equally. We report test results as compared to a Bonferroni corrected significance value (α = 0.0001), where α is divided by the number of tests—96—before comparison.

**Overview Analysis 2: Can sonic features be predicted from combined gesture features?**

We hypothesize that sonic features can be predicted from kinematic features, with head and hand features combined being more predictive than simply one or the other. To investigate this, we train a Gradient Boosting regressor on all six kinematic features to predict each individual sonic feature (four models in total). A grid search was performed over the following hyperparameters: n_estimators ∈ {50, 100, 150, 200}, learning_rate ∈ {0.001, 0.01, 0.1}, and max_depth ∈ {2, 4, 8, 10}. We select models based on repeated three‐fold cross‐validation with five repetitions and stratified sampling to balance classes. We evaluate our model at training time using the *R* ^2^ score and report our results on a holdout subset of the DTW distance dataframe corresponding to 20% of the entire dataset (not used at all during training).

We further investigate the effect of looking only at either head or hand kinematic features, repeating the process four times: once on randomized data, once on the kinematic features corresponding to the head, once on the kinematic features corresponding to the hand, and once on both hand/head features. As before, we also repeat this analysis on subsets of the DTW distance dataframe corresponding to individual performers.

## 3 Results

**Analysis 1 results: Do sonic motif DTW distances covary with spatiotemporal patterns of gesture?**

**Fig. 3:**
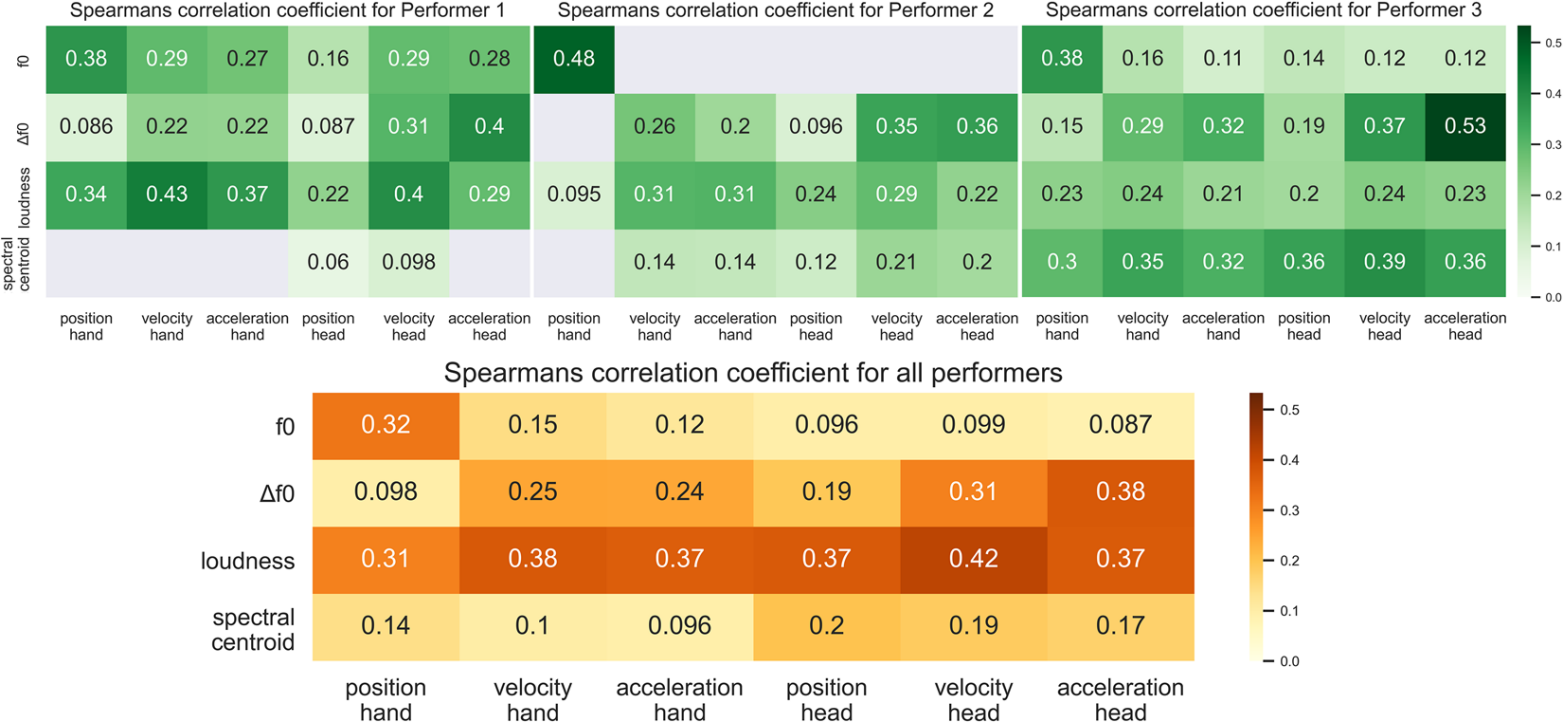
Spearman's correlation coefficient for each sonic and kinematic feature. Absent squares represent tests with a *p*‐value above the Bonferroni corrected significance level of (.0001/96). It can be seen that each performer has distinct patterns of correlation strengths. A full table of results is provided in the Supplementary Materials, Table S2.

Across all performers and performances, we find a significant positive correlation between all kinematic distances and all sonic distances (up to .42 *r*’s, *p*s < .0001). For individual performers, these correlations are greater (up to .53 *r*’s, *p*s < .0001), with notable individual differences observed. Fig. 3 shows these correlation coefficients in the form of a heatmap. Nonsignificant correlations (i.e., *p* > .0001) are excluded from the heatmaps and displayed as gray squares.

Overall, loudness correlates most strongly across the kinematic features, with head velocity being most prominent (*r* = .42), followed closely by all other kinematic features (.31 < *r* < .38). The relationship between the kinematic features and spectral centroid is much weaker (.096 < *r* < .20), driven largely by Performer 3, with other performers exhibiting either no or very weak correlation.

We observe a notable overall relationship between pitch and the position of the hand (*r* = .32). Among individual performers, this is most pronounced for Performer 2 (*r* = .48), but evident across all performers (.38 < *r* < .48). However, with the exception of Performer 1, the relationship between pitch and all other kinematic features is considerably weaker. Of the two pitch‐related features, it is in fact with Δf0 that we observe the strongest and most consistent relationship with kinematics. The correlation magnitudes and order of importance between Δf0 and each of the kinematic features is consistent across all performers, the strongest of which is with head velocity/acceleration (.31 < *r* < .53), followed closely by hand velocity/acceleration (.22 < *r* < .32) and finally, more weakly with head/hand position (.096 < *r* < .19).

To ensure that the observed correlations are not due to spurious chance‐covariance between kinematic and sonic features across all motifs within performers, we repeat Analysis 1 on the same dataset with DTW values shuffled randomly within features and performer sets, that is, DTW values are shuffled within each type of feature and remain attributed to the same performer as in the original dataset. In this randomized test, none of the 96 individual tests yields a significant result (*ps* > .0001/96), nor a single correlation (|*r*|*s* < .04), as compared to 85 significant test results in the original unshuffled data. The mean and standard deviations of the *p*‐values corresponding to the 96 real and randomized tests are as follows: *M_real_* = 0.0175, *SD_real_* = 0.0986, *M_random_* = 0.493, *SD_random_* = 0.299. We can, therefore, conclude that gestures that are randomly paired with vocal motifs do not co‐structure with vocal acoustics. This test demonstrates that gesture‐vocal co‐structuring does not occur simply because they are performed by the same vocalist, but rather that gesture and vocal motifs co‐structure along the dimensions we explore (kinematic and sonic features) that characterize the gesture and vocal events.

**Analysis 2 results: Can sonic features be predicted from combined gesture features?**

Given that each performer has their own co‐structuring profile, relating to constraints of movement and vocalization, we seek to learn whether and how the combined kinematic features can predict the sonic features.

**Fig. 4:**
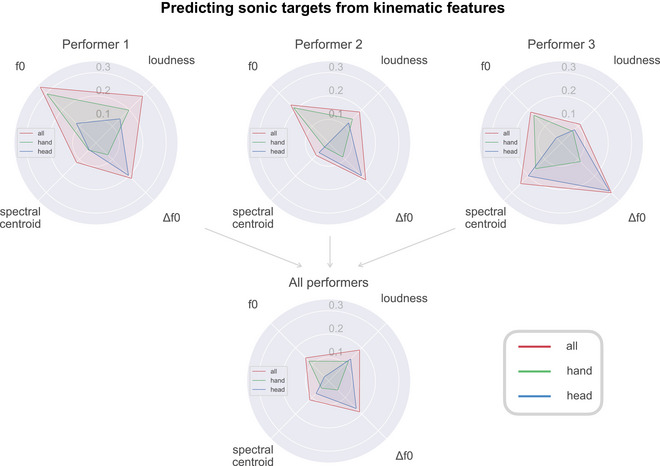
A visualization of test *R* ^2^ values for regression models trained on kinematic features (hands only, head only, and “all” combined) to predict each sonic target for individual and all performers. It can be seen that the combined head and hand kinematic features have a higher *R* ^2^ for predicting a single sonic feature than either head or hand alone. A table of numerical results can be found in the Supplementary Materials, Table S3.

Across all performers and performances, we observe a significantly better than chance prediction of all individual sonic features when using the combined kinematic features; Fig. 4 shows the *R* ^2^ values for these regression models on the test dataset. For models trained on all kinematic features, the most predictable sonic features overall are Δf0 and loudness (both *R* ^2^ = .19), followed closely by f0 (*R* ^2^ = .14) and spectral centroid (*R* ^2^ = .11). On a performer level, we observe greater values for the pitch‐based sonic features: f0 (.19 < *R* ^2^ < .34), Δf0 (.21 < *R* ^2^ < .30), and more variation for loudness (.11 < *R* ^2^ < .28) and spectral centroid (.08 < *R* ^2^ < .25). Overall, and for each of the individual performers, we notice that the predictive power of all head and hand kinematic features combined surpasses either the head or hand features when considered alone. In the randomized test for Analysis 2, none of the models trained on the shuffled data demonstrated any predictive power (−.05 < *R* ^2^ < .05).

Based on the predictive power of kinematics regarding sonic features, as seen in the results of Analysis 2, we conclude that kinematics hold information about vocal motifs across all four sonic dimensions. This provides further evidence that co‐structuring of vocal features is occurring with respect to both manual and head motions.

### 3.1 Dynamic visualization pipeline

A key implication of the current work is that performers each have their own co‐structuring profile that captures, to some extent, the overall qualities of their multimodal performances. As such, we believe that our analyses that describe these profiles can also contribute to a qualitative investigation of multimodal performance from a musicological perspective. For this purpose, intuitive dynamic dashboards can be valuable, where quantitative static data points are linked with the original dynamic data (Miao et al., 2025). We, therefore, offer animations of the performances with an integrated dashboard that visualizes the DTW distance dataframe using dimensionality reduction techniques: https://thomasgnuttall.github.io/KarnatakGestureVocalCostructuring.

#### 3.1.1 Animations

Animations were created by exporting FBX files of the motion capture data recorded using the Xsens Awinda system. These FBX file data were then retargeted to a human base mesh using 3DS Max, cloth simulation was created in Marvelous Designer, and rendered using Unreal Engine.

#### 3.1.2 Dashboard

**Fig. 5:**
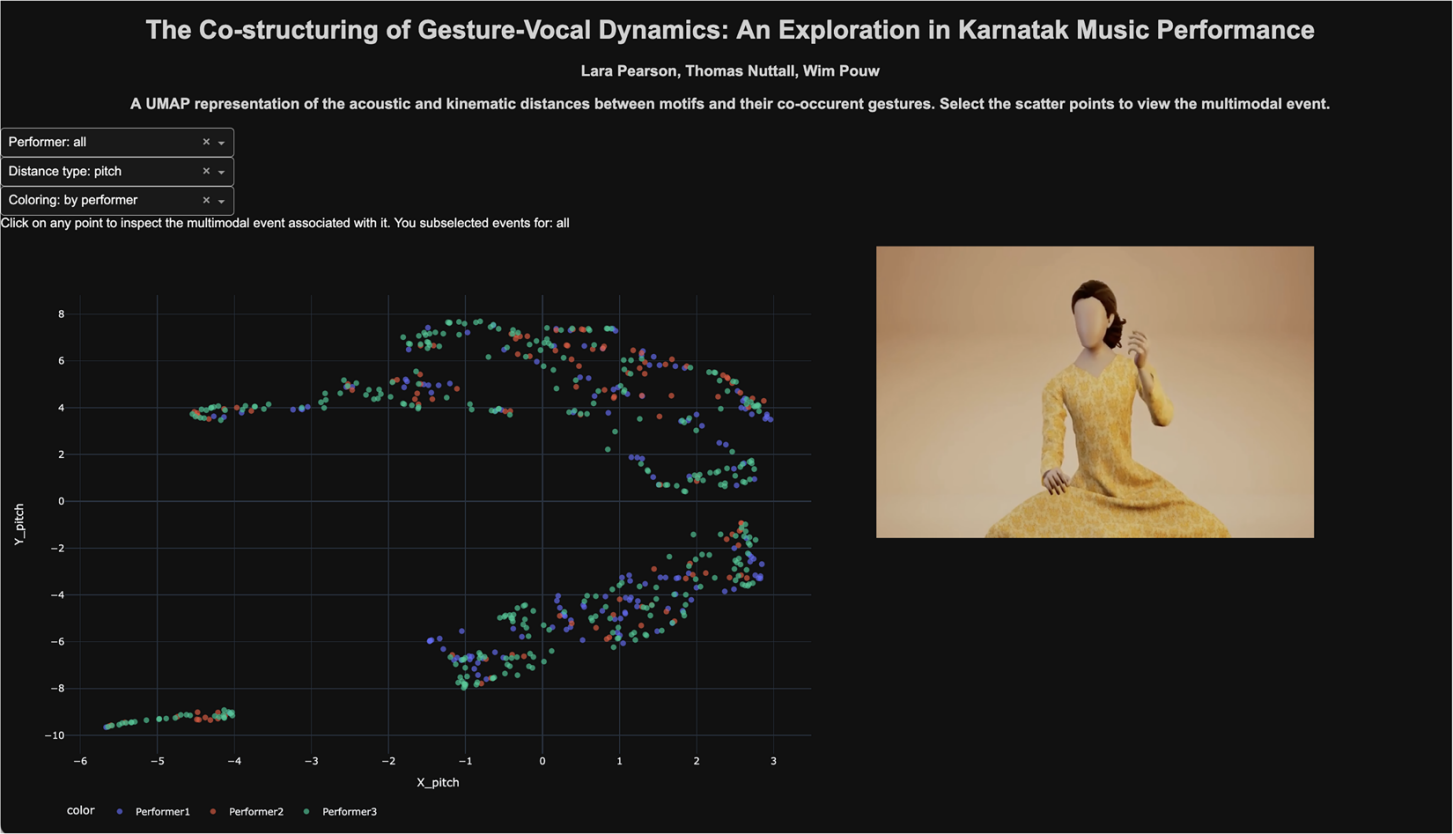
A dynamic dashboard for data exploration. The interface of the dashboard is shown. Users can click on any point in the 2D embedding space and set filters to adjust whether all performers or only one performer is shown, and whether to color the points by performer, raga, or performance, as well as set the variable of interest (e.g., pitch [f0] or hand position). When clicking on a point, the animated audiovisual recording of that gesture‐motif is shown. By clicking on nearby points, the user can explore highly similar gesture‐motifs, and by looking at other regions or clusters in the space, the user is able to assess those that are more dissimilar, which can be cross‐checked visually by inspecting the animations.

In Python, using Plotly Dash, we developed a dashboard that links the animated audiovisual recordings of each identified motif with a UMAP (Uniform Manifold Approximation and Projection) representation of the gesture kinematic distances and sonic distances. This application allows users to identify whether similar vocal motifs also have similar gestures, and vice versa. The application can be used for further explorations of possible structural combinations of kinematic and sonic features in this dataset (e.g., performer/performance clusters, unique gestures, dimension‐specific gestures). The dashboard (see Fig. 5) can thus be used as an exploratory hypotheses‐generating tool that will increase the usability of the current dataset, which is also made open access with this publication as a contribution. We host the application on an Apache2‐supported server, which will be online indefinitely: https://thomasgnuttall.github.io/KarnatakGestureVocalCostructuring. The code for recreating the dashboard and running it locally can be found in the accompanying GitHub repository.

#### 3.1.3 Data preparation for the dashboard

We used R to prepare the datasets as input for the dashboard. In the DTW distance dataframe, for each motif (*N* = 595) we have DTW distances for each sonic and kinematic variable (e.g., loudness, hand position, etc.) relative to every other motif. In essence, we have a high‐dimensional NxN embedding space where each motif has a location in that space, which is defined relative to the (dis)similarity of all other motifs. For visualization purposes, we can represent this high‐dimensional embedding space on a 2D plane using dimensionality reduction. We used the R package “UMAP” for this. The resulting output is x, y coordinates for each motif, where points closer in space are more similar. It should be noted that 2D representations of high‐dimensional spaces are often distorted and may not properly reflect the actual global structure of the data. This is why we do not perform statistics on dimensionality‐reduced data with UMAP, and instead use it as a tool to visualize highly similar sonic or co‐vocal gestures for all the motifs in an efficient way.

## 4 Discussion

The results of our analyses show that sound and body movement are systematically related at the motif level, which suggests the potential for multimodal meaning‐formation through co‐structuring. We note that within this dataset, the various sonic and kinematic features examined differ in the extent to which they co‐structure; loudness correlates most strongly across the kinematic features, with notable co‐structuring also between pitch (f0) and hand position. However, of the two pitch‐related features, it is the change in pitch (Δf0) that has the strongest and most consistent co‐structuring with kinematics across all performers. We see that individual performers reliably co‐structure sound and movement using differing characteristic salient dimensions (e.g., prioritizing co‐structuring between pitch and hand position, or change in pitch and head acceleration). The regression results demonstrate that sound−gesture relationships are better understood when hand and head motion is combined, indicating a more whole‐body coordination with vocalization. In sum, it is clear from our investigation that performers co‐structure head and hand gestures with vocalizations, and they exploit different dimensions of possible couplings, which reflects the rich variability between performers that determines their multimodal style.

As vocal motifs that are more alike are found to co‐occur with gestures that are more alike, this suggests that gestures are constrained by the vocal motifs with which they are produced. There may also be constraints in the other direction—gesture constraining vocalization—but as music performance is fundamentally organized and structured through sonic features (vocal phrases), it appears more likely that gestures become recruited within that organization rather than the other way around. The co‐structuring found, therefore, suggests that the degrees of freedom of gesture are constrained and organized relative to the vocal modality. This reduction of the degrees of freedom is statistically evident from our analyses, which demonstrate that randomly paired gesture kinematics do not show a co‐structuring with the vocal modality, and only gestures that occur under similar motifs show statistically reliable kinematic similarities.

Bernstein (1967) famously argued that the nervous system does not play the body like keys on a piano, such that for each modality, or for each muscle (or for each muscle spindle, for that matter), there is an independent control to achieve a certain task. This would make for a biologically intractable control system. Rather, depending on the particular task, there are so‐called synergies, where subsystems’ degrees of freedom are locked in relation to each other, and some subsystems are allowed to vary more freely. The nervous system is, therefore, in the business of organizing into task‐dependent synergies. We speculate that the reduction of the degrees of freedom of gesture and vocalization in this example of Karnatak vocal performance is a task‐dependent synergy, where the task is defined by aesthetic and articulatory performance variables; performance variables that are in any case more complex than the original Bernsteinian example of hammering a nail (Bernstein, 1967; for a discussion, see Latash, 2008, Chapter 8 on meaning as the performance variable). This speculation aligns with work by researchers who aim to extend the concept of synergy, and explicate the types of performance variables we are dealing with in the social and artistic performative ecological niche. For example, Alviar, Kello, and Dale (2023) talk about pragmatic modes in communication, which are multimodal synergies that can span multiple signal modalities, time scales, and dimensions. Davidson (2008) also underlines in the context of music performances the multidimensionality of the performance variable(s),^4^ which can be understood as defining synergies.

While we think our findings are in line with such a broader construct of synergies, our analyses do not directly investigate the synergy as such. For example, we do not know what happens to the vocal modality if we manipulate gesture, or if we perturb the gesture trajectory, so as to ascertain a truly synergistic relationship between gesture and vocalization. We also do not conduct an Uncontrolled Manifold Analysis (Latash, 2008), where one would assess how some performance variable (e.g., execution of a vocal motif) is stabilized by maintaining a dynamic but constant relationship between gesture and vocalization, while allowing for some task‐irrelevant variation. As such, we see our findings as a necessary but not sufficient indication of a reduction of degrees of freedom. Whether such reductions are due to functional relationships that serve the task goal is a matter of further experimentation, if not a matter of further explanation of what exactly is controlled in music performance and communicative practices (Latash, 2008, Chapter 8).

### 4.1 Individual performer profiles of co‐structuring

It can be seen from Fig. 3 that each performer has their own particular co‐structuring profile. The strongest correlations for Performer 1 are between loudness and head/hand movement, with a few other correlations at similar levels, for example, between head acceleration and change in pitch. While for Performer 1, the stronger correlations are, therefore, quite broadly distributed across features, for Performer 2, there is a clearer strongest correlation between pitch and hand position. Performer 3 also has a clear, strongest correlation, but in this case, between pitch and head acceleration. The regression analyses (see Fig. 4) highlight some of the same relationships: for example, showing that Performer 3 is the only one for whom kinematics are predictive of spectral centroid. The regression analyses also show that, in general, head movement is more predictive of change in pitch, while hand movement is more predictive of pitch.

This study thereby opens up opportunities for perception studies (e.g., Huang et al., 2017; Luck et al., 2010; Morrison et al., 2014; Trujillo et al., 2018) as they relate to co‐structuring profiles. For example, it can be asked whether particular performer‐specific co‐structuring dimensions, as well as the overall dimensionality of the co‐structuring profile (how diverse vs. uniform is the profile in terms of co‐structuring values across the dimensions), resonate with audience members’ experience of the performance: for instance, in relation to any emotions conveyed (Livingstone & Palmer, 2016). This analytical approach is, of course, not limited to music making, and the pipeline can be applied in behavioral biology to assess the potential mate‐selection consequences of multimodal performances in birds (Soma & Shibata, 2023), or the audience's experience of public speaking performances (Chollet & Scherer, 2017).

We hope that the methods used in the current article provide a more multidimensional characterization of multimodal performances that can be used as a basis to understand how such co‐structuring profiles relate to the expressive and semiotic qualities of different performances and performers. We can see from watching vocalists perform that they have different gesturing styles; the analytical approaches presented here provide insight into why this is the case, and allow a quantification of where those differences lie. The analyses also help us understand which features of the musical sound are more strongly indexed by performers (either individually or across individuals) through co‐structuring of gesture and sound, which has semiotic implications for the performance and indeed for the musical style. With the opening of the current dataset and a dashboard made for data exploration, we invite further research on these topics.

### 4.2 Stereotypical gesture‐vocalization co‐occurrence

While it is possible to observe in our dataset recurring gestural forms that co‐occur with particular types of melodic movement, we argue that both these and the larger array of gesture‐vocalizations found in performances are not sufficiently characterized by the idea of isolated sound‐gesture units that are structurally combined from a mental library of stereotypical gesture types. In this section, we discuss why, with reference to our results above.

Recurrent gestures observed through qualitative analysis of the performances in our dataset include small circular hand motions co‐occurring with oscillating ornaments known as *kampita* (see https://youtu.be/FKoucCIcmtM) and two‐handed stretching motions co‐occurring with a range of moderately emphatic melodic movements, often involving ornaments that briefly touch on a higher pitch before pulling down onto a lower pitch (see https://youtu.be/9zQd17uSQdY). To better understand vocalists’ production of these two recurring gestures, using manual annotation in ELAN video annotation software (Lausberg & Sloetjes, 2009), we analyzed their appearance in a subset of the dataset, comprising two ragas (Anandabhairavi and Atana) each performed by the three performers: a total of six performances (results are provided in the Supplementary Materials, Table S4). The analysis reveals great variability in the way that these recurring gestures are used across performers and performances. Some vocalists produce these recurring gestures more than others. For example, Performer 1 often uses the two‐handed stretching gestures (34 times across the two performances), but the gesture is entirely absent in Performers 2 and 3. Instead, for motifs where Performer 1 uses two‐handed stretching gestures, Performer 3 often uses a hand gesture that pushes out toward the audience and then back toward his body (see https://youtu.be/ky0uQXINAWY).

Even within performances by a single performer, such recurring gestures do not have a one‐to‐one relationship to particular motifs. Stretching gestures made by Performer 1 in raga Anandabhairavi can be seen accompanying a wide array of somewhat emphatic melodic movements, across at least 10 different motifs. Therefore, in these performances, any stable meaning, such as should be apparent in a recurrent gesture (Müller, 2018), appears to be broader than a particular motif, indicating instead a more general melodic/sonic quality, such as emphasis or oscillation. Meanwhile, the same performer may produce the same melodic movement with either a stretching gesture or an entirely different hand gesture. For example, in a performance of raga Atana, Performer 1 uses the stretching gesture five times in the first 30 s, and then abandons it completely for the remainder of the performance, even when singing the motifs with which the stretching gesture originally co‐occurred. Finally, the borders between definitions of recurring gestures can be fuzzy, for example, the “circular” gesture that co‐occurs with oscillating melodic movement tends to look like a circle in Performer 1 (see https://youtu.be/FKoucCIcmtM), but in Performer 3, it appears more as a repeated pulsing or pushing motion, without much circular trajectory (e.g., https://youtu.be/sW4LQUAF9xA).

Due to these issues discussed above, we suggest that the gesture‐vocal coherence that an audience and performer might experience is best understood as a continuous co‐structuring, where some aspects are allowed to vary, while other degrees of freedom are more co‐constrained for a particular expressive quality or idea. Thus, while it might seem evident that there are undeniable cases of gesture‐vocal co‐occurrence, there is also more continuous coherence that cannot be clearly expressed in categorical terms (e.g., circle, stretch) and is better characterized in terms of co‐structuring profiles, such as those we present in this article.

The analyses in this study reveal individual performers’ co‐structuring profiles expressed as correlations between dimensions. These profiles provide insight into individual performers’ gesturing habits, which, in addition to tendencies toward producing stronger relationships between specific gestural and sonic dimensions, might also include, for example, a tendency to produce, or not produce, two‐handed stretching gestures. The recurring gestures observed (stretching and circular hand motions) are both the results of constraints (bodily, musical, and sociocultural) as well as acting themselves as constraints on what the performer is likely to produce at any given moment, due to their habitual aspect. Individual bodily differences will also likely affect gesturing habits (Caldeira et al., 2021), as such physical differences will affect biomechanic co‐stabilities of vocal production and upper body movement (Pearson & Pouw, 2022; Pouw & Fuchs, 2022). Other important constraints include performers’ ideation of the music while they sing (which may bring into play either crossmodal perceptual correspondences or expressivity relating to mood or emotion), their expressive and aesthetic goals in relation to the audience, and also their music learning and life experience (e.g., the impact of learning over a period of many years from a teacher who gestures in particular ways). All of these constraints act upon the performer in the moment, resulting in particular profiles of gesture‐vocal co‐structuring.

### 4.3 Limitations of the research and future directions

One feature of this study that may be considered a limitation in certain musicological contexts is that the motifs found using the machine learning approach are not always segmented at points that would likely be chosen by an expert human annotator. This is the result of the process used, which prioritizes the identification of pairwise combinations of regions of high similarity, with no explicit information regarding what might constitute a boundary considered musically meaningful by an expert. As our analyses are based on measurements of similarity (DTW), we argue that using vocal motifs that were originally found based on an automated assessment of similarity is ideal. It means that our vocal motif dataset includes a diverse range of motifs, each of which has at least one highly similar match. This then provides a good comparison with all of the other vocal motifs that are either somewhat similar or entirely dissimilar. However, as a result of this process, some of the motifs would not be considered whole from a musicological perspective. To improve motif segmentation, either an updated automated approach or manual annotations could be considered for future research. It would be interesting to learn whether using manually chosen motifs has any effect on the results reported here. We would expect that using only a small number of different manually chosen motifs could give results that are not typical of the wider performance: for example, if the chosen vocal motifs are associated with stereotypical gestures. However, if a very wide range of different manually segmented motifs were chosen, it is an open question whether the results would differ to those in this study. It should be noted that although motifs are meaningful in this style, in manual annotation studies, expert musicians may nevertheless disagree on motif boundaries (Pearson & Manickavasakan, 2023). Therefore, with expert annotations, although the borders will often be more plausible from a musicological perspective, they will still differ across different instances of the same or highly similar melodic material.

The study is based on a large number of motifs (*n* = 595) from 31 different performances given by three expert Karnatak vocalists. Due to the small sample size of different vocalists, we would caution against generalizing the findings of this study to the whole population of Karnatak vocalists. However, the vocalists were chosen carefully for qualities aimed at ensuring they are representative of proficient Karnatak vocalists based in South India; They (a) have a high level of expertise, (b) are active and successful in the Karnatak music concert scene in South India, and (c) studied with well‐known teachers who are acknowledged experts. At the same time, we ensured some diversity across the tradition by choosing performers who come from different teaching lineages and do not typically perform with each other. It should be noted that while there has been a lot of emphasis on increasing participant numbers in cognitive science, issues of reproducibility lie equally (and sometimes more) in the number of samples that are taken within participants (Brysbaert, 2019); it is also about sample size, and not only about the number of participants. Our dataset consists of 44 performances, 31 of which are represented by the 595 automatically found motifs, which constitutes many within‐subject gesture‐vocal samples. While the low number of participants limits the generalizability to a theoretical “population” of Karnatak performers, the many repeated measures of our study ensure that the effect observed is likely present in these individuals, and is likely to reproduce with this group. Future research could focus more on generalizability and collect more data from different performers. Furthermore, future studies can further explore how performers’ gesture‐vocal co‐structuring profiles change over key periods of time, such as during their training or early stages of their performing careers. This would provide insight into how individuals’ gesturing styles develop, and the possible influences on this development.

### 4.4 The semiotic potential of gesture‐vocal co‐structuring

It is worth considering the semiotic potential of the co‐structuring found in this study—what meaning hand and head gestures may be able to convey regarding the music being sung. Existing studies on gesture and vocalization have, for example, found that head movements hold information regarding vocalized pitch (Yehia et al., 2002) and that this can be picked up to some extent by viewers (Thompson et al., 2010). Furthermore, it has been found that the length of a percussionist's performance gesture can affect the perception of tone duration (Schutz & Manning 2012), and seeing co‐speech gestures can influence the perception of prosodic stress (Bosker & Peeters 2021), showing that gestures can, in combination with sound, influence perception and also meaning as a result. Indeed, ethnographic research with Karnatak and Hindustani vocalists suggests that in their experience, co‐singing gesture can convey information regarding musical qualities, including pitch movement, emphasis, and articulation (Fatone et al., 2011; Pearson, 2016; Rahaim, 2012). In the current study, we find co‐structuring across a range of kinematic and sonic dimensions, and show that sonic features (f0, Δf0, loudness, and spectral centroid) could be predicted better than chance from the combined kinematic features. We know that at least some of this co‐structuring is due to direct coupling because of findings in Pearson and Pouw (2022), which used an overlapping dataset and found coupling, for example, between peaks in hand velocity/acceleration and change in pitch. The relevant semiotic relation in such direct coupling would be contiguity, where meaning is formed through systematic bordering (spatial and/or temporal) of one thing against another (Mittelberg & Hinnell, 2023), in this case, the bordering between change in pitch and change in hand velocity/acceleration. However, because the current study examines a second‐order relation (an association between associations), the co‐structuring observed may go beyond direct coupling (see Fig. 1). The semiotic potential of the co‐structuring found may equally be symbolic, in the sense that if someone is repeatedly exposed to the use of similar gestures when uttering similar phrases, even if the two do not directly couple in their peaks or trajectories, at some point, the two will start to indicate each other (Mittelberg, 2008).^5^ Although it is clear from our regression analysis that the combined kinematic features hold some information regarding sonic features, we have not examined this from a perception perspective. Future research could test whether listeners can determine which gesture fits with which vocal motif, either after becoming familiar with the style of the performer, or without any familiarity at all. If listeners can determine the gesture that actually occurred with the vocal motif, it would indicate that some kind of co‐structuring relation has been perceptually learned (when familiar with the performer), or acquired early on by virtue of sharing a similar human body that has similar gesture‐vocal stabilities (Pouw & Dixon, 2022; Pouw & Fuchs, 2022; Sheets‐Johnstone, 2011). Going further, future experimental work could also be based on artificially created gestures that manipulate part of the systematicity present in the found co‐structuring profiles, for example, to assess learnability.

## 5 Conclusions

It has been argued that meaning in multimodal language can be understood by analyzing the neighboring context, much like how methods in natural language processing can glean semantic information from text using the principle of distributional semantics (Boleda, 2020). In some performance‐based styles, such as the present Karnatak music context, gesture and vocalization appear entangled to an even greater extent than in everyday co‐speech gesturing. Here, we show that there is indeed a continuous co‐structuring of gesture‐vocal performances at multiple dimensions. We suggest that this multidimensional co‐structuring profile captures the style of the performer, and when viewed across performers, provides insight into the multimodal semiotic potential of the musical performance style more broadly. We thereby contribute to the wider project of understanding multimodal meaning‐making.

## Open Research Badges

This article has earned Open Data and Open Materials badges. Data and Materials are available at https://osf.io/6huvd/ and https://github.com/thomasgnuttall/KarnatakGestureVocalCostructuring/tree/main.

## Supporting information



Supporting Information

## Notes

1 Gesture is defined in this article as visible bodily motion tied to utterance (Kendon, 2004). The gesturing of Karnatak vocalists appears as a flow of upper body movement, which includes, for example, tracing, pointing, pulling, pushing, flicking, and holding motions.

2 The dataset can be found at https://osf.io/6huvd/.

3
https://github.com/thomasgnuttall/KarnatakGestureVocalCostructuring/tree/main

4 “The movements used to generate these stimuli draw from individual movement repertoires, some of which reveal intimate personal states, others of which are for much more direct communicative ends, e.g., end the phrase now!” (Davidson, 2008, p. 374).

5 “Contrary to emblems, most spontaneously produced gestures cannot said to be, in strictly Peircean terms, inherently and predominantly symbolic. Nonetheless, here, too, a gradual habituation due to recurring gesture‐meaning pairings in specific environments can result in an increased degree of symbolization and eventually in a new repertoire of conventionalized, i.e., symbolic forms” (Mittelberg, 2008, p. 130).
