# Supplementary material for: The Co‐Structuring of Gesture‐Vocal Dynamics: An Exploration in Karnatak Music Performance
Source: Cogn Sci. 2025 Nov 30;49(11):e70137. doi: 10.1111/cogs.70137 (PMC12665335; doi:10.1111/cogs.70137)
Supplement: Supplementary file 1 — Supporting Information [file COGS-49-e70137-s001.docx]

**Supplemental materials for “The co-structuring of gesture-vocal dynamics: An exploration in Karnatak music performance”**

**Section S1. Pattern Finding**

The pattern finding model is presented in (Nuttall, Plaja-Roglans, Pearson, & Serra, 2022) and the implementation we use is found in the compIAM tools repository (Plaja-Roglans, Nuttall, & Serra, 2023). The steps used for extracting the motifs are not a contribution of the current study, and can be found in this published notebook:

<https://mtg.github.io/IAM-tutorial-ismir22/melodic_analysis/melodic-pattern-discovery.html> (Nuttall, Plaja-Roglans, Pearson, Manickavasakan, et al., 2022).

We applied this method to our 44 performance recordings from 3 vocalists and the model found 595 motifs from across 31 of the performances (see Table S1 for full list of motifs found per performer, raga, and recording session).


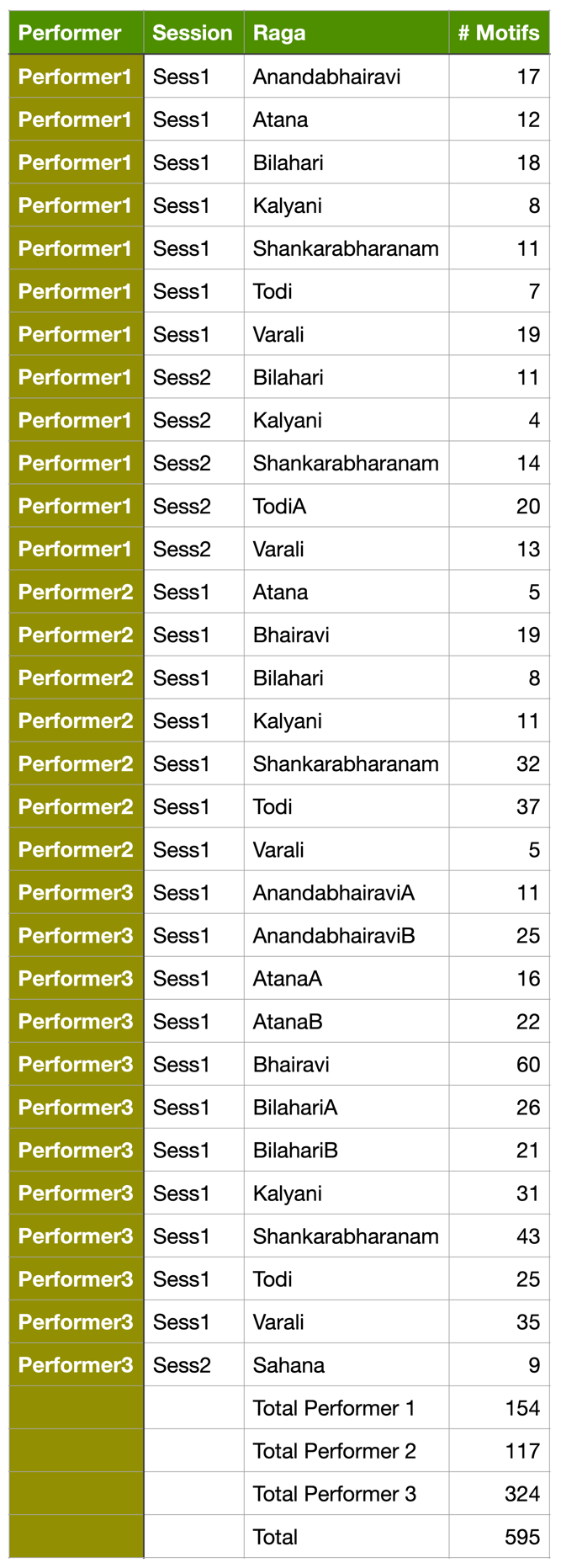


**Table S1:** Total number of motifs found per performer, raga, and recording session. For analyses involving multiple performers, the data is subsampled so as to ensure an equal number of motifs from each performer.

**Section S2. Pitch Time Series**

The pitch time series are extracted using the FTA-NET carnatic model in the compIAM library (Plaja-Roglans, Nuttall, & Serra, 2023). The derivative of these curves (and the subsequent kinematic curves) is estimated using the average of the slope of the line through the point in question and its left neighbor, and the slope of the line through the left neighbor and the right neighbor (Keogh & Pazzani, 2001). Pitch time series are smoothed using a 1-dimensional gaussian filter with sigma=2.5, decided by manual inspection of the pitch curves and audio.

Silences of 350ms or less are interpolated to account for small silences present due to consonant sounds or glottal stops - in such cases there is arguably a real break in pitch (due to, for example, a glottal stop) however this break often does not correspond to a real conceptual break in the phrase being sang by the performer. Since we subsequently use DTW on these pitch curves, which does not allow for breaks of any length within the analysed time series, interpolation ensures their inclusion.

**Section S3. Spectral Centroid**

Spectral centroid is computed as the "center of mass" of the frequency spectrum i.e. the average frequency of the spectrum at each time step, weighted by energy in that frequency bin. The frequency spectrum is computed using a short term fourier transform with parameters: window size=2048, hop_length=512, window=hanning.

**Section S4. Kinematic Time Series**

We normalize the position curves to exist in the same space by calculating the position centroid of the left and right shoulder for each performance, computing the angle between the line joining these two centroids and the x axis (which is fixed to magnetic north (© Xsens Technologies B.V., 2018)). We rotate the gesture data such that the line between shoulder centroids is parallel to the x axis, to ensure the performer is facing “front of stage”. We get the pelvis centroid for each performance from the original mocap data and change the origin of the kinematic feature space to correspond to that centroid. For the head gesture series, the “height” of the performer is computed as the distance between the pelvis centroid and the head centroid. Each head position vector is divided by height so as to account for differences in performer height.

Kinematic time series are smoothed using a second-order Savitzky Golay filter with window length 125ms, decided by manual inspection of the curves alongside the video.

**Section S5. Identifying the dominant hand**

To identify the performer's dominant hand we compute the kinetic energy for each individual motif using the velocity data for the left and right hand. Kinetic energy can be computed from velocity, v, and mass, m, using KE = (mv^2)/2. m/2 is a constant and can be removed since we are looking at which of the two values is larger (=v^2). We assign a “dominant hand” tag to each motif, corresponding to which hand had the higher kinetic energy in that instance.

For our analysis we use the gesture data from the dominant hand, and mirror where necessary to ensure that all motifs appear to exist in the same space.

88.57% are identified as Left handed

11.43% are identified as Right handed

The ratio in energy between the identified dominant hand and non-dominant hand is:

<energy of the dominant hand> / <energy of the non-dominant hand>

The proportion of motifs with a ratio greater than 1 is 100.0%

The proportion of motifs with a ratio greater than 1.2 is 97.48%

The proportion of motifs with a ratio greater than 2 is 91.09%

The proportion of motifs with a ratio greater than 10 is 87.23%

The proportion of motifs with a ratio greater than 100 is 73.28%

Therefore, the dominant hand for each motif tends to be very clear.

**Section S6. Dynamic Time Warping Metric**

The dynamic time warping distance metric is computed using a Sakoe-chiba radius (warping window) equal in length to 10% of the length of the longest of the two patterns compared. This was decided based on the following process. The Karnatak musicologist amongst the authors first labelled 50 pairs of motifs that would be considered the same in a musical context, and then checked which of a range of possible warping radiuses provided results where these “same motif” annotations achieved the lowest DTW distances. We then made manual inspection of a sample of time series pairs, ensuring that desirable time series information (such as the melodic movement in ornamentations known as *gamaka*) was not lost due to a too generous window, whilst still allowing for slight time dilation between two instances of the same pattern.

To account for slight differences in start/end segmentation points between two occurrences of the same pattern, the warping path starts at the two points with the lowest Euclidean distance within this warping window from the start of the compared patterns, likewise the warping path ends at the two closest points within one warping window distance from the end of the compared patterns.

**Section S7. Relationship Between Dynamic Time Warping and Perceived Melodic Similarity**

In addition to checking qualitatively that both the sonic and kinematic similarity indicated by DTW distance aligned with our human perception of similarity (as described in Section S6 above), we also conducted a more systematic check for melodic similarity. To validate the use of DTW as a proxy for melodic similarity between pitch motifs we first asked a professional Karnatak vocalist to annotate the 800 most similar motif pairs in the DTW distance dataframe (lowest 800 DTW values between f0 curves). This vocalist was a performer in our study, but had not previously heard the motifs in isolation or seen any of the musicologists’ same/different annotations. The vocalist was presented with the audios of each motif pair and asked to label them as “same” or “different”. Definitions were provided to the vocalist beforehand (see Box S1).

| ***Same*** *-the two audio clips are highly similar, defined as melodically at least 80% the same. There may, however, be some fleeting pitch differences (e.g., gamaka differences), minor start and end point differences, or differences in loudness or speed. If sa is placed on a different pitch but the clips are otherwise the same, the pair should also be considered in this ‘same’ category.*    ***Different*** *- the two are either entirely different, or that less than 80% of one audio clip is found in the other (less than 80% the same). This may sometimes be difficult to assess. If it seems to be a borderline case, please place it in the ‘same’ category.* |
| --- |

**Box S1:** Definitions provided to the professional Karnatak vocalist for the same/different annotation process. Feedback from the vocalist indicated that when using these definitions, the majority of motif pairs were clearly either the same or different, but that there were a number of motif pairs (25 out of 800) where it was more difficult to decide.


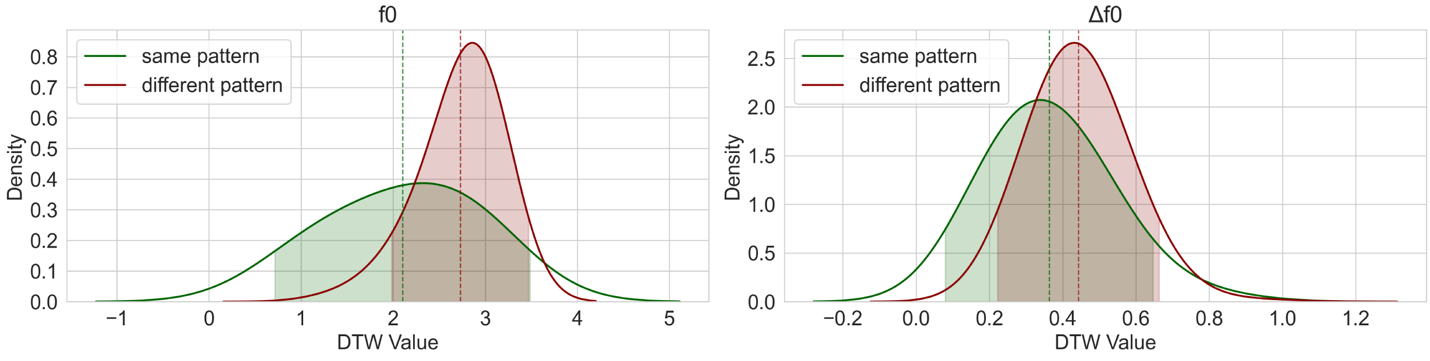


**Figure S1**: The plots display the kernel density estimates of DTW distances for the f0 and Δf0 time series corresponding to motif pairs in the “same” and “different” groups.

We perform independent t-tests to compare the means of the same and different samples for both f0 **(***M_different_* = 2.73, *SD_different_* = 0.38; *M_same_* = 2.10, *SD_same_* = 0.72) and Δf0 **(***M_different_* = 0.44, *SD_different_* = 0.11; *M_same_* = 0.36, *SD_same_* = 0.15). For f0, we obtain a statistically significant difference between same and differently categorised motifs, *t* (798) = 15.84, *p* < .0001. A statistical difference was also found for Δf0, *t* (798) = 8.05, *p* < .0001 (see Figure S1 for plots displaying the kernel density estimates). Furthermore, we compute the point biserial correlation coefficient, *r_pb_* between the DTW distances and the categorical label of “same” and “different”: for f0 **(***r_pb_* **=** 0.49, p < .0001) and Δf0 **(***r_pb_* **=** 0.28, p < .0001).

We validated the same/different labels made by the first Karnatak vocalist annotator with a second Karnatak vocalist annotator who was not a performer in this study and who had over 15 years of performance experience. This second annotator performed the same perceptual test for 400 of the top 800 labels. The inter-annotator agreement between the two annotators was 92.79%. The confusion matrix of agreement between the two is shown in Figure S2.


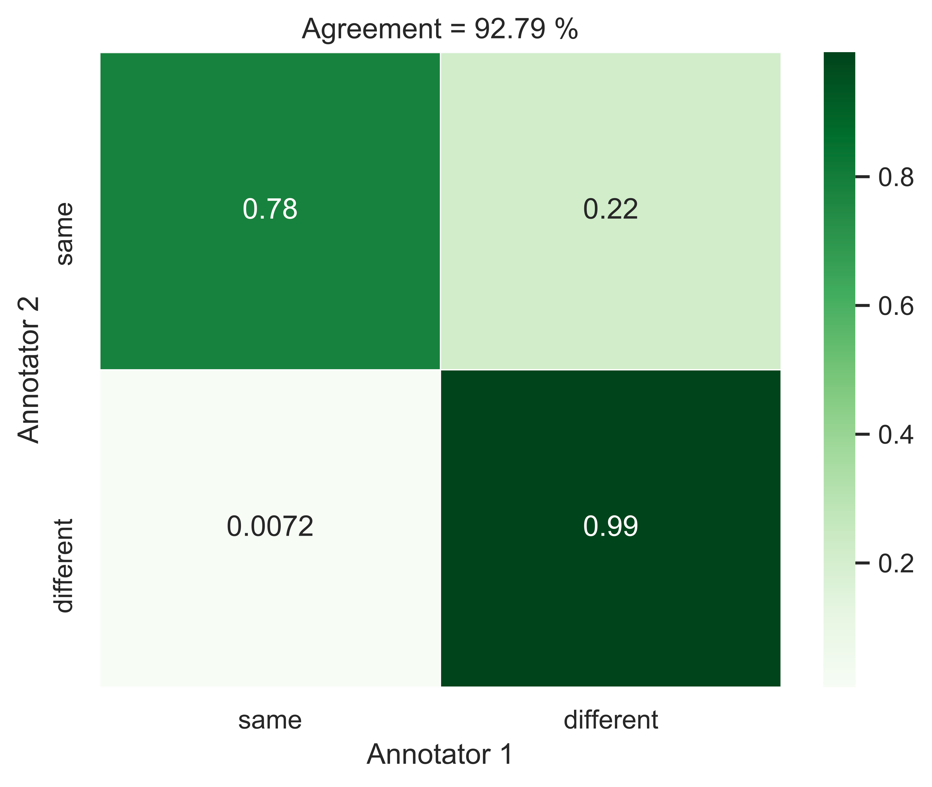


**Figure S2**: Inter-annotator agreement between the two Karnatak vocalist annotators. Measured as the proportion of the 400 mutually performed tests labelled with the same label.

We conclude that although DTW distance does not fully align with the expert categorization of same and different motifs, the distributions are sufficiently distinct to warrant using DTW as a proxy for melodic similarity. It should be noted that assessments of this type made by an expert musician will be coloured by their knowledge of the style. For example, two highly similar pitch curves that are audibly in different ragas (melodic types) are likely to be defined as different by an expert musician, notwithstanding their high degree of melodic similarity. Such subtleties may contribute to the overlap seen in the results.

**Section S8: Tables**


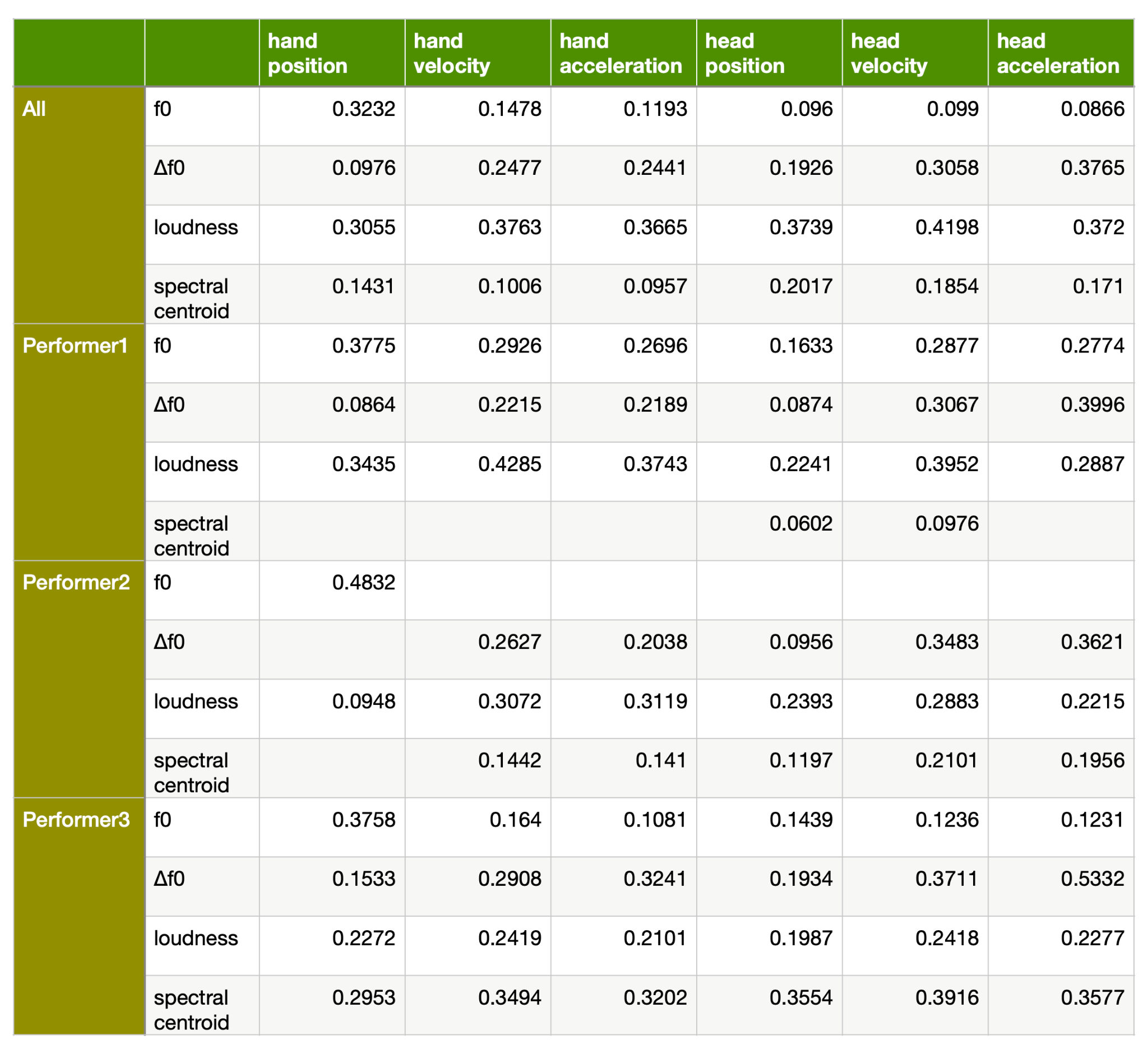


**Table S2:** Spearman’s correlation coefficient for each sonic and kinematic feature. Empty cells represent tests with *p*-value above the Bonferroni corrected significance level of 0.0001/96. Sample sizes are as follows: All, 61 425; Performer 1, 11781; Performer 2, 6786; Performer 3, 52326


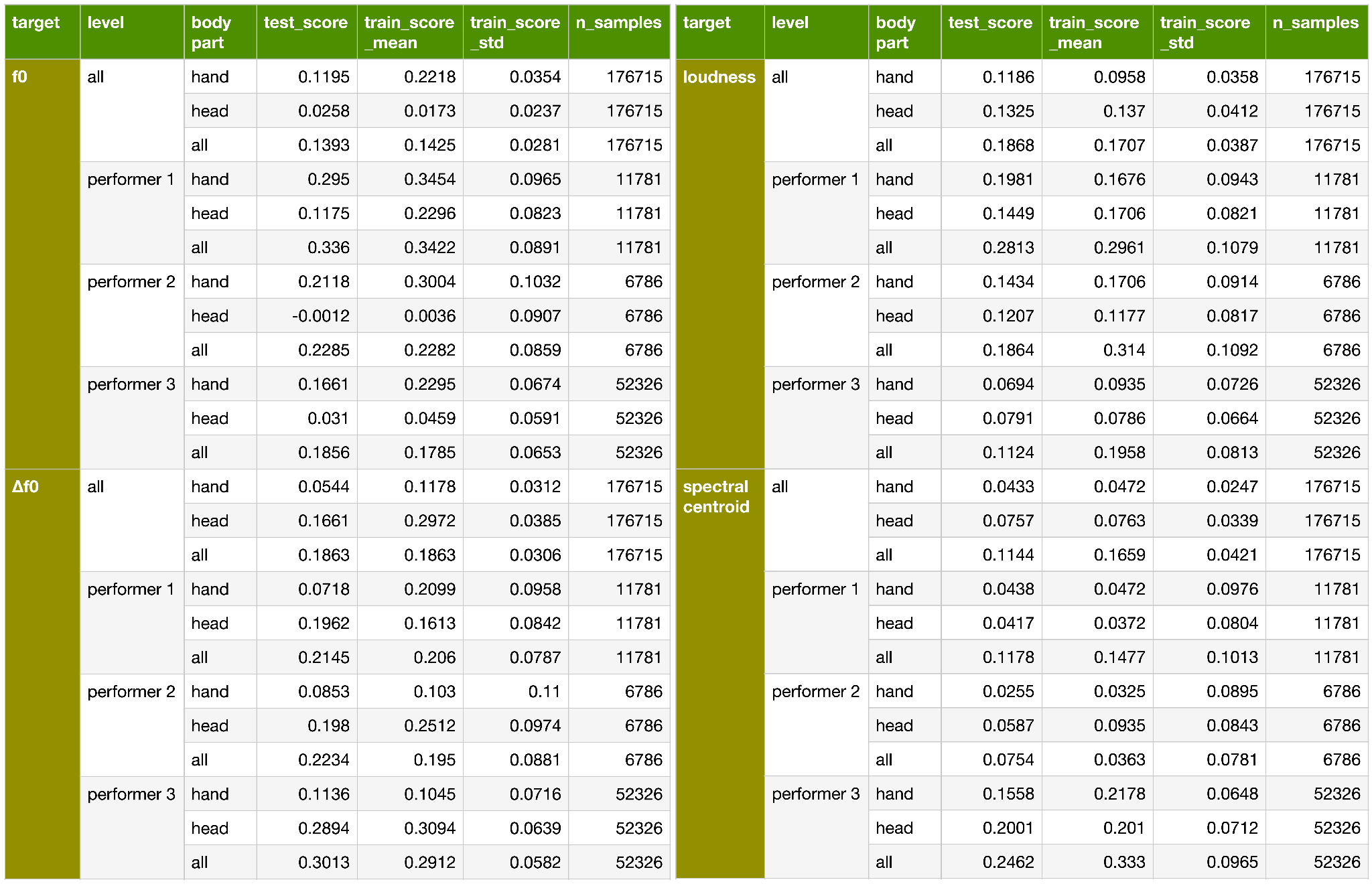


**Table S3:** *R*^2^ values for regression models trained on kinematic features to predict each sonic target. *test_score* refers to the result on an unseen test set, and *train_score_mean*/*train_score_std* refers to the mean and standard deviation of R2 across all folds in the repeated k-fold cross validation.


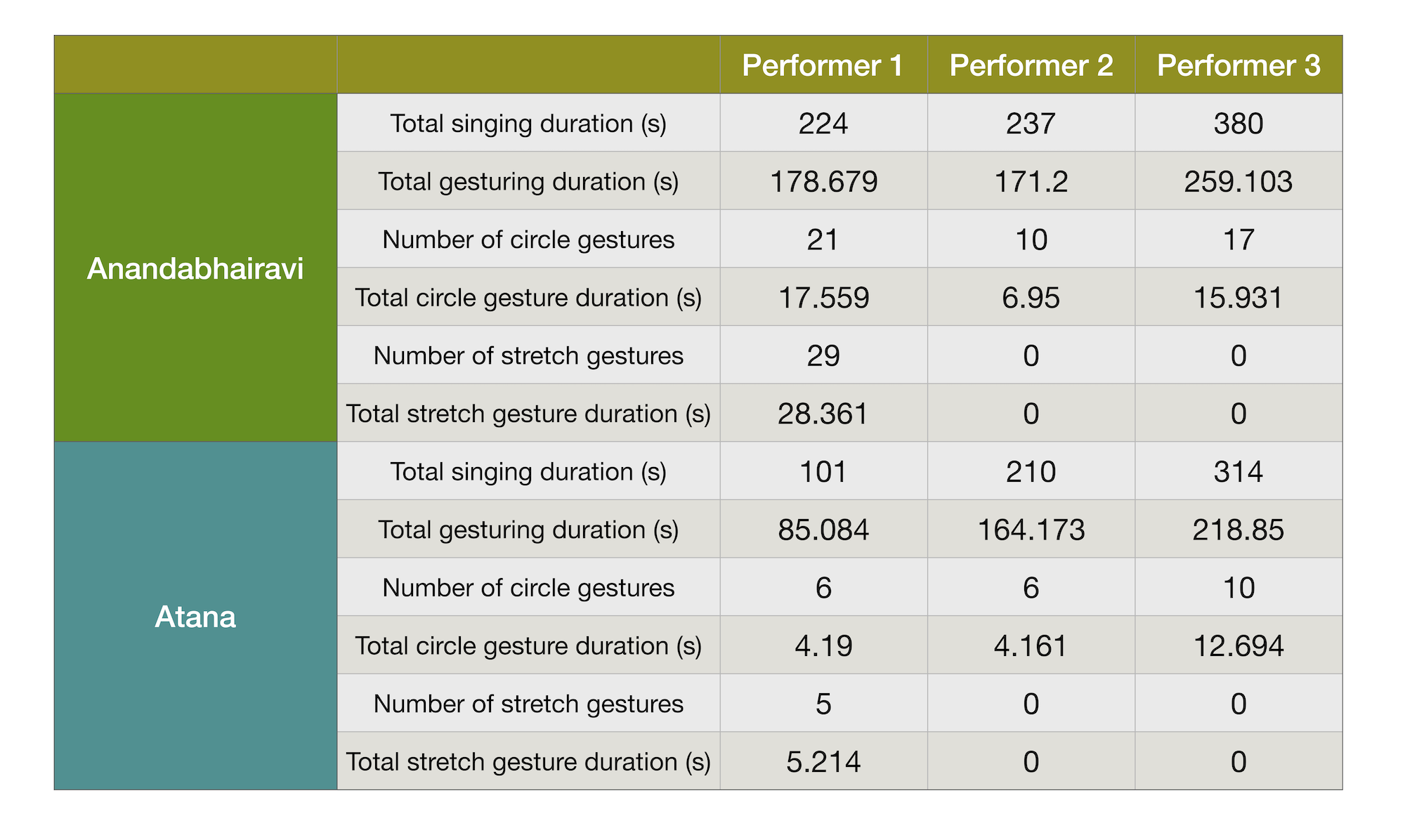


**Table S4:** Recurring gesture analysis results. Circle gestures were defined as small repeating circular or pulsing hand gestures. Stretch gestures were defined as a two-handed gesture where the hands start together and then pull apart, as though stretching something.
